# Regulation of the yeast metabolic cycle by transcription factors with periodic activities

**DOI:** 10.1186/1752-0509-5-160

**Published:** 2011-10-12

**Authors:** Aliz R Rao, Matteo Pellegrini

**Affiliations:** 1 Bioinformatics Interdepartmental Program, University of California, Los Angeles, USA; 2 Department of Molecular, Cell, and Developmental Biology, University of California, Los Angeles, USA

## Abstract

**Background:**

When growing budding yeast under continuous, nutrient-limited conditions, over half of yeast genes exhibit periodic expression patterns. Periodicity can also be observed in respiration, in the timing of cell division, as well as in various metabolite levels. Knowing the transcription factors involved in the yeast metabolic cycle is helpful for determining the cascade of regulatory events that cause these patterns.

**Results:**

Transcription factor activities were estimated by linear regression using time series and genome-wide transcription factor binding data. Time-translation matrices were estimated using least squares and were used to model the interactions between the most significant transcription factors. The top transcription factors have functions involving respiration, cell cycle events, amino acid metabolism and glycolysis. Key regulators of transitions between phases of the yeast metabolic cycle appear to be Hap1, Hap4, Gcn4, Msn4, Swi6 and Adr1.

**Conclusions:**

Analysis of the phases at which transcription factor activities peak supports previous findings suggesting that the various cellular functions occur during specific phases of the yeast metabolic cycle.

## Background

Budding yeast cells (*Saccharomyces cerevisiae*) exhibit oscillatory dynamics in several cellular pathways, such as those involving the cell cycle, glucose metabolism, and respiration. Previous studies [1,2] have observed metabolic cycles in yeast cultures in which most genes were expressed in a cyclic manner. These cycles are self-sustaining oscillatory patterns: once cells are synchronized, they continue to exhibit robust oscillations indefinitely [3,4]. Microarray analysis revealed that cells exhibit cycles consisting of long reductive phases and short respiratory bursts, accompanied by corresponding changes in dissolved oxygen levels, and cell cycle events are restricted to the reductive phase [1,2]. Interestingly, the periods of the yeast metabolic cycles vary in the two studies. Klevecz *et al*. [1] reports a period of ~40 mins, and Tu *et al*. [2] observed periods of ~300 mins. There is debate about the relationship between the cell division cycle and the yeast respiratory oscillations, and whether the short- and long-phase oscillations are fundamentally different, or whether the variation in phase length is a result of a difference in nutrient availability, cell spacing, or errors in data interpretation [5-7].

Analysis of the regulatory network of transcription factors involved in the genomewide oscillations may shed light on the underlying causes of the yeast metabolic cycle. A previous study suggests that the Cbf1-Met4-Met28-Met31-Met32 transcription regulatory complex and Gcn4p are important in regulating the short-period metabolic cycle, although it is not likely that there is a single pathway responsible for the observed oscillation. Rather, there are several coupled subsystems involved, with no hierarchical control [8]. The underlying metabolite responsible for synchrony in cells seems to be hydrogen sulfide [9]. There are no previous studies aimed specifically at determining the transcription factors regulating the long-period yeast metabolic cycle, although Lelandais *et al*. [10] proposes that the transcription factors Hap1, which is heme-activated and is known to function as an oxygen-sensor, and Hap4, a subunit of a heme-activated complex, may be important. Since each long-period cycle is characterized by the upregulation of several clusters of genes with different functions in the various phases, there is no doubt there are more key transcription factors regulating the timing of cellular events. The goal of this study is to reveal information about the network of transcription factors regulating the longer (~300 min) metabolic cycle.

The long-period yeast metabolic cycle consists of three phases: Ox (oxidative, respiratory), R/B (reductive, building) and R/C (reductive, charging). Each phase is associated with a characteristic change in dissolved oxygen levels in the yeast culture. During the Ox phase, oxygen levels drop drastically. In the R/B phase, oxygen levels increase, while in the R/C phase, the longest of the three, oxygen levels stay relatively constant. During the course of the experiment, the yeast culture is continuously infused with low levels of glucose, however glucose levels in the media are almost zero at all phases of the cycle; cells appear to adsorb and metabolize available glucose immediately. Analyses of microarray time series expression data revealed that ~57% of yeast genes exhibit periodic expression during the course of a metabolic cycle and cluster into one the three superclusters, corresponding to the three phases of the yeast metabolic cycle. Gene expression in different clusters peaks at different phases, and many common metabolites also oscillate, indicating that there is a clear temporal separation between various cellular events [2,11].

In the oxidative phase, oxygen is rapidly consumed in a burst of respiration. Genes whose expression peaks during this phase are highly expressed during a very narrow window of the yeast metabolic cycle. Functional and metabolome analysis indicates that in the Ox phase, oxidative phosphorylation is using up previously accumulated acetyl-CoA while ATP is being rapidly produced. The oxidative cluster is enriched for genes involving amino acid synthesis and ribosomes, indicating that cells are preparing for cell division. Genes involved in sulfur metabolism and RNA metabolism also show increased expression. During the Ox phase, ATP is abundant, and this is what enables the assembly of translation machinery for the next phase: the reductive/building phase [2,12].

In the R/B phase, 40-50% of cells enter the cell cycle during each cycle of the yeast metabolic cycle [2]. Therefore, expression increases for genes involved in cell division. Examples of these are histone genes, spindle pole genes, and genes involved in DNA replication. Meanwhile, respiration is shut off, possibly to protect DNA from oxidative damage during cell division. Instead, yeast cells shift to glycolysis and fermentation. Oxygen consumption ceases, and mitochondria are rebuilt. Consequently, the R/B cluster is also enriched for genes involving mitochondrial biogenesis [2,12].

Finally, in the R/C phase, cells become dependent on non-respiratory modes of metabolism, and acetyl-CoA accumulates, which is a precursor to the upcoming respiratory Ox phase. The R/C cluster is enriched for genes involving fatty acid oxidation, glycolysis, stress-associated response and protein degradation, and this also includes genes involved in peroxisomal function, vacuoles and ubiquination machinery. Little oxygen is being consumed, and dissolved oxygen levels continue to rise. Altogether, cycles in metabolism, respiration and mitochondrial function are all important components of the yeast metabolic cycle [2,12].

Analysis of intracellular concentrations of metabolites shows that many metabolites show periodic oscillations during the yeast metabolic cycle, and some may be important in the establishment and regulation of cycles [11]. NADP(H), sulfur and heme metabolic pathways may be especially important, and blocking the production of either of these metabolites prevents oscillations from appearing [11].

Time-series microarray data may be analyzed to determine the transcription factors that are most likely regulating the periodic genes. Other studies searched the promoters of periodic genes to find the most frequently occurring motifs and deduce the most significant transcription factors [8,10]. Cokus *et al*. [13] developed an alternative method to reverse-engineer the regulatory network behind oscillating cellular systems. For each transcription factor, linear regression is used to calculate *α*-coefficients, which capture whether the genes a transcription factor binds to are differentially expressed or not, assuming that the effects of other transcription factors are held constant. They are essentially a measure of transcription factor activity, and when calculated for each time point, one can find the *α*-coefficients ("activities") over time for each transcription factor.

Transcription factor *α*-coefficient profiles can be further analyzed for periodicity, and treated as if they were time-series expression data. Cokus *et al*. [13] uses a Fourier-based periodogram method to find the most periodic transcription factors, under the assumption that the transcription factors exhibiting the most robust oscillations are the ones likely to be regulating the cycle involved. Fourier analysis works well for scoring *α*-coefficient profiles that resemble a sinusoidal curve. However, it would not be as effective for studying expression profiles from the yeast metabolic cycle, because some clusters of genes contain a sharp spike or two peaks per cycle [2] (Supporting Material). In this case, calculating the autocorrelation function would give a more accurate periodicity score. Since the length of each period is known, we expect to see a peak in a specific location, and the relative magnitude of the peak is a measure of periodicity.

In order to determine the connections between the transcription factors themselves, Cokus *et al*. [13] used the collection of time-dependent *α*-coefficients to compute a time-translation matrix. This can be used to determine *α*-coefficients for successive time points using matrix multiplication, and allows one to compute the asymptotic amplitudes and phases of the transcription factor *α*-coefficients, as well. Since each entry of the time-translation matrix can be interpreted as one transcription factor affecting the *α*-coefficient of another in the successive time point, this is a useful quantitative model of the dynamical properties of the system.

The amplitudes and phases of the *α*-coefficients may also be estimated directly from the *α*-coefficient profiles, instead of using the translation matrix. Lelandais *et al*. [10] developed a Bayesian decomposition based algorithm, EDPM, to decompose gene expression profiles into a sum of predefined model patterns: sine waves with equivalent periods but different phases. Thus, each time-series profile is represented as the sum of sine waves with various phases and amplitudes, and the magnitudes of the contributing model patterns are a unique "footprint" for each profile. From these, the best-fitting phases and amplitudes can be calculated for each gene or, for the purposes of this study, each transcription factor.

We analyzed time-series microarray data from a previous study [2] to identify the transcription factors regulating the yeast metabolic cycle. We used the methods of Cokus *et al*. [13] to calculate transcription factor *α*-coefficients using linear regression. We also calculated the time-translation matrix, with some modifications from the previous study's methods. Considering that oxygen is a major oscillating metabolite, and that Hap1, a likely candidate for metabolic cycle regulation, had been previously described to function as an oxygen sensor [14], we also included oxygen in the transition matrix, and hence, in the dynamical model. *α*-coefficient profiles were analyzed for periodicity using autocorrelation. Finally, the phase and amplitude of each transcription factor's *α*-coefficients were calculated using a simplified version of the EDPM algorithm [10]. Essentially, for each transcription factor, we found the single sine wave that best fits its *α*-coefficient profile.

The advantage of the linear regression based method for estimating transcription factor activities is that calculations use existing high-throughput data to provide an elegant, purely computational solution for finding not only the most periodic and most robustly oscillating transcription factors, but also the network of relationships between them. The goal of this study is to identify the key transcription factors regulating the yeast metabolic cycle and to construct a dynamical model of the activities of these transcription factors. The hypothesis is that the most significant transcription factors encompass cellular functions corresponding to the known phases of the yeast metabolic cycle as previously defined in [2], [12].

## Results

### Transcription factors with highly periodic *α*-coefficients

We selected transcription factors that had significant *α*-coefficients at a large number of time points, and iteratively recomputed *α*-coefficients and p-values to obtain a reduced set of transcription factors. Among these, we selected the transcription factors with periodic *α*-coefficients. The 13 transcription factors that remain after iterative selection and filtering for high periodicity include the expected transcription factors Hap1, Hap4 and Gcn4 (Table 1). None of the transcription factors in the Cbf1-Met4-Met28-Met31-Met32 complex, important in regulating short-period metabolic oscillations in yeast, are found in the list. Neither is any transcription factor with an explicit role in sulfur metabolism. About half of the transcription factors are known cell cycle regulators. This is not surprising, since roughly 50% of yeast cells in the culture divide in each cycle, and cell division is always initiated within a short window during the yeast metabolic cycle, in the R/B phase [2].

**Table 1:** List of top 13 transcription factors

TF	Periodicity	Phase	Amplitude	Function
**ACE2**	0.520	45°	0.048	Cell cycle (early G1 specific transcription).
**ADR1**	0.518	48°	0.047	Glucose repression.
**ARO80**	0.544	44°	0.066	Aromatic AA degradation
**BAS1**	0.562	293°	0.082	Recombination. Purine and histidine synthesis.
**GAL3**	0.452	215°	0.036	Galactose metabolism.
**GCN4**	0.548	2°	0.085	Main regulator of general AA control.
**HAP1**	0.449	80°	0.049	Respiration. Heme-responsive. Growth potential.
**HAP3**	0.503	255°	0.048	Respiration. Subunit of heme-activ'd Hap2/3/4/5.
**HAP4**	0.620	227°	0.124	Respiration. Subunit of heme-activ'd Hap2/3/4/5.
**MSN4**	0.595	0°	0.062	Cell cycle. Stress-responsive gene expression.
**RLR1**	0.515	42°	0.063	Recombination. Transcriptional elongation.
**SPT2**	0.497	302°	0.108	Cell cycle (interacts w/histones, SWI-SNF).
**SWI6**	0.631	225°	0.058	Cell cycle (progression from G1 to S phase).

Shown are the transcription factors that remain after iterative filtering for the highest periodicity scores of their α-coefficients. Phase and amplitude were calculated from the best-fit sine waves.

**Figure 1:**
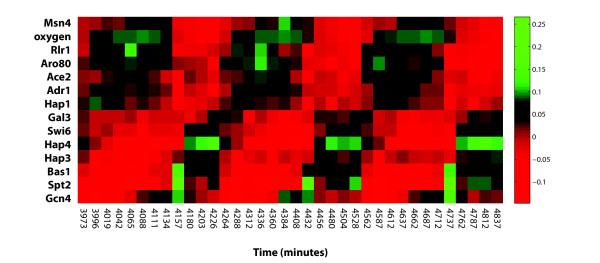
**α-coefficients of transcription factors regulating yeast metabolic cycle**. A heat map of the α-coefficients of the top transcription factors, at each time point in the dataset. Factors have been ordered by peak α-coefficient values. Oxygen levels from Tu *et al*. [2] have also been normalized and included in the figure.

Ordering transcription factors according to phase reveals a clear temporal separation between peaks in their *α*-coefficients (Figure 1). A spike in several transcription factors can be seen at the time points corresponding to 4157, 4432 and 4737 minutes. Specifically, there is a brief increase in *α*-coefficients for transcription factors Bas1, Spt2 and Gcn4, and a decrease for Ace2, Adr1 and Hap1 (Figure 1). This coincides with the time at which oxygen levels are dropping at the fastest rate. Normalized oxygen levels were also included in Figure 1 for comparison.

**Figure 2:**
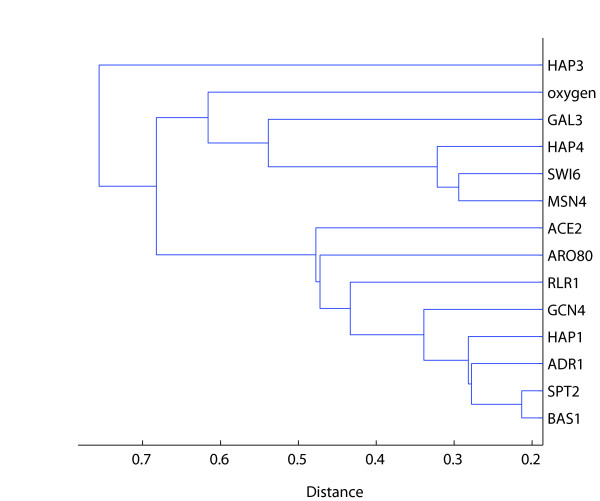
**Clustering results using absolute values of α-coefficients**. Many transcription factors cluster closely with factors that have α-coefficient curves that are approximately negatives of each other (Figure 1).

**Figure 3:**
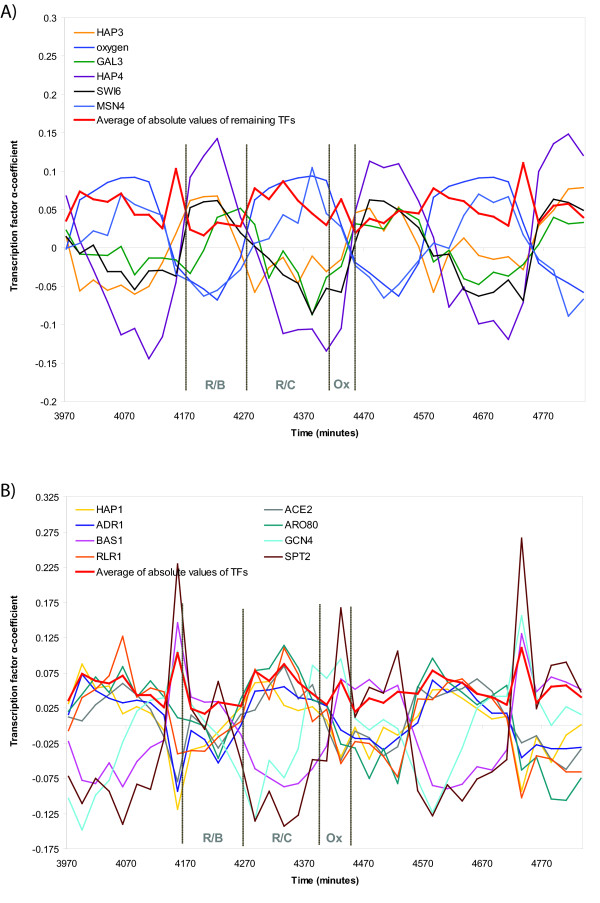
**α-coefficients of transcription factors regulating yeast metabolic cycle**. A) shows all transcription factors. The 8 transcription factors in the largest cluster are represented as a single curve equal to the average of the absolute values of their α-coefficients. B) separates the transcription factor α-coefficients contributing to the averaged curve.

Cluster analysis reveals that the transcription factors exhibiting sharp spikes belong to the same cluster (Figure 2). In fact, most of the transcription factors have similarly shaped profiles, with the exception of the five transcription factors Hap3, Hap4, Gal3, Msn4, and Swi6 (Figure 2, 3). The two distinct clusters of transcription factor profiles are shown separately in Figure 3, which presents a different view of the heatmap in Figure 1. Oxygen levels do not closely follow any curve, although the curve appears most similar to the opposites of Hap3 and Gal3 *α*-coefficients (Figure 1, 2, 3).

**Figure 4:**
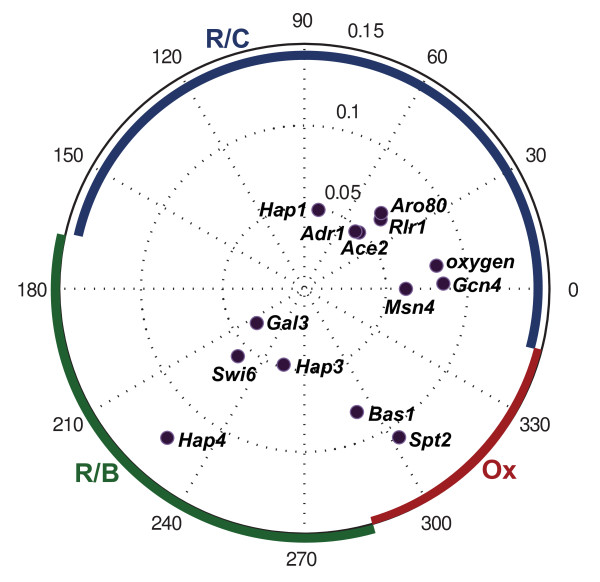
**Amplitudes and phases of transcription factor α-coefficients, with phases of the best-fit-sine wave**. Amplitudes are those of the best-fit sine waves.

The phases and amplitudes of the best-fit sine waves for the *α*-coefficients are shown in Figure 4. Note that transcription factors with similar curves that would otherwise cluster together have a phase shift of 180° relative to one another if the curves are opposite.

**Figure 5:**
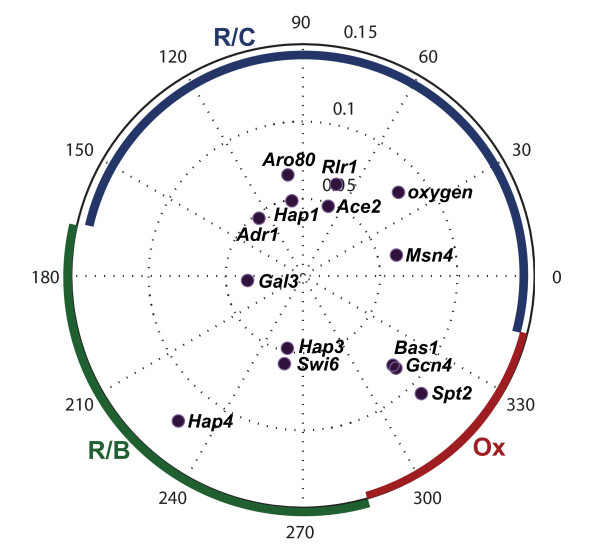
**Amplitudes and phases of transcription factor α-coefficients, according to the maximum α-coefficient value**. The peaks were calculated from average α-coefficient curves, averaged over three metabolic cycles. Amplitudes are the same as in Figure 4, those of the best-fit sine waves.

The three phases of the metabolic cycle are defined by the changes in oxygen levels [2], and we can use this to determine what phase different transcription factor *α*-coefficient profiles peak in. In the Ox phase, dissolved oxygen levels drop dramatically. The R/B phase is characterized by no oxygen consumption in the yeast cells, i.e. dissolved oxygen levels increase. The remaining phase is the R/C phase. Using this classification, the temporal location of the phases of the metabolic cycle can be assigned on Figure 3 and aligned with the points on the polar plot in Figures 4 and 5. Thus, had we calculated best-fit cosine waves instead of sine waves, the points would represent peaks in oscillations, and the Ox phase would be around 285-345°, the R/B phase would be around 165-285°, and the R/C phase, which is the longest of the three [12], would occupy the area around -15-165° (Figures 4, 5). We expect transcription factor activities to peak in different phases according to their cellular function. For example, Gcn4, the main regulator of amino acid control, should peak in the Ox phase, and Swi6, a transcription factor involved in cell cycle initiation, should peak near the beginning of the R/B phase. The phases of the best fit sine waves do not fit expectations for every transcription factor (Figure 4). Instead, taking the maximum of the transcription factor *α*-coefficients generates results that are more consistent with the known function of these factors (Figure 5).

### Regulatory network among transcription factors

**Figure 6:**
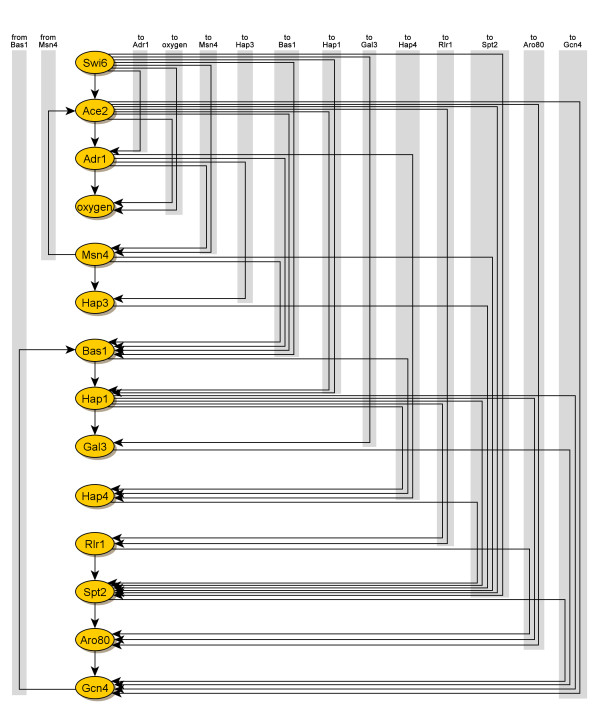
**Network of transcription factors regulating the yeast metabolic cycle**. The time-translation matrix is illustrated as a dynamical model of the α-coefficients of the 13 most significant transcription factors, in which the matrix entries of highest absolute values represent couplings between transcription factors. A line from factor *A *to factor *B *indicates that the activity of *A *affects the activity of *B *at the next time point. The displayed order of factors minimizes the number of upward arcs (these arcs being grouped on the right side of the figure). This representation can be seen in [13]. Levels of dissolved oxygen in the yeast culture were normalized and included in the dynamical model.

A transition matrix was calculated to model the network of relationships between transcription factors. A previous study used a transition matrix with entries constrained to be positive [13], but our results indicate that the unconstrained transition matrix is a better representation of this system. When the matrix was constrained for non-negative entries, the most significant entries corresponded to the most significant positive entries in the non-constrained matrix, with the difference that significant negative entries were close to zero (Additional Files 1 and 2). Upon closer observation of the non-constrained time-translation matrix, transcription factors with opposite *α*-coefficients, such as Bas1, Spt2 and Gcn4 compared to the opposite group Ace2, Adr1 and Hap1, have column entries resembling the opposite groups in absolute value but with opposing signs. Assuming that the two opposite clusters are indeed closely related in regulating the yeast metabolic cycle, the large negative entries should also count as significant relationships, since they would be significant *positive *entries, had the *α*-coefficient curves been mirrored across the x-axis. Calculations of the model residuals confirms that the non-constrained transition matrix models the regulatory network more accurately (Additional File 3). For this reason, the nonconstrained transition matrix was used when illustrating the regulatory network graphically. If an entry was greater than 0.5 in absolute value, the connection between transcription factors was deemed significant. This threshold resulted in a connected graph with only a small number of transcription factor pairs that are connected in both directions. Finally, nodes were arranged to maximize the number of downward arrows (Figure 6).

## Discussion

The transcription factors that we identify as regulators of the yeast metabolic cycle peak can be classified into the three phases described by Tu *et al*. [2]. The transcription factor Swi6, a component of the SWI-SNF complex that is responsible for the progression of the cell cycle from G1 to S phase, peaks in the R/B phase. This is consistent with the fact that many genes involved in the cell cycle peak in the R/B phase [2]. We expect to find that transcription factors involved in stress-responsive adaptation, glycolysis, and protein degradation peak in the R/C phase. Msn4, which is involved in stress-responsive gene expression, peaks in this phase, and so does Aro80, a transcription factor involved in the degradation of aromatic amino acids. Adr1, a transcription factor involved in glucose metabolism, peaks in the R/C phase. Amino acid synthesis and RNA metabolism takes place mainly in the Ox phase, and this is consistent with the peaks for a transcription factor involving general amino acid control, Gcn4, and a regulator of RNA polyadenylation and transcription elongation, Spt2. Some transcription factors may have similar functions and yet peak in different phases. For example, Hap1 is involved in respiration, and so are Hap3 and Hap4, but Hap1 clearly peaks in the R/C phase, while the two members of the Hap2/3/4/5 complex are most active in the R/B phase. For the most part, however, transcription factor *α*-coefficients peak in the phases that we would predict based on their known functions. Many of the transcription factors are known to be associated with pathways that may be related to phases or transitions in the yeast metabolic cycle, such as the diauxic shift, response to oxidative stress, or cell cycle initiation. Examples of these transcription factors are Hap1, Hap4, Swi6 and Msn4.

### Hap4

This transcription factor has been previously suggested to have an important role in regulating the yeast metabolic cycle [10] and is the regulatory subunit of the Hap2/3/4/5 complex [15]. It functions as a transcriptional regulator of mitochondrial function by regulating genes involved in the respiratory pathway [15,16], and it is also an important regulator of mitochondrial biogenesis and allows mitochondrial function to escape from glucose repression [15,17]. The transcription factor Hap4 responds primarily to the availability of carbon source, and secondarily to heme [15].

### Hap1

Another key regulator of respiration in yeast, Hap1, is directly regulated by heme, an intermediate in the signaling mechanism for oxygen levels in yeast [18]. In aerobic growth conditions, when oxygen levels are high, increased heme levels activate Hap1 [14,19]. Then, Hap1 promotes the transcription of many genes encoding functions involving respiration and the control of oxidative damage [19,20]. As a result, we see that in the Ox phase of the yeast metabolic cycle, when oxygen levels drop sharply, Hap1 also shows a sudden decrease in activity. This drop in Hap1 activity marks a decrease in the rate of respiration after that point, and the rate of oxygen consumption appears to level off afterwards, as yeast cells enter the R/B phase. Interestingly, Hap1 activity appears to follow the derivative of the curve for dissolved oxygen levels (Figure 3A/B), i.e. it is proportional to the change in the amount of oxygen. Hap1 acts as an activator in aerobic conditions and a repressor in hypoxic conditions, which likely allows for greater transcriptional control in response to changing oxygen levels [21].

### Swi6

The transcription factor with the most periodic activity during the yeast metabolic cycle is Swi6, a known regulator of the yeast cell cycle [22-26]. Therefore, this transcription factor is likely to be the one synchronizing cell division initiation in the R/B phase. The question then becomes, what is regulating Swi6 activity, and what triggers cell division initiation? The high levels of ATP produced during respiration cannot be the inducing signal for progression into the S phase, because glucose-starved cells can contain significant levels of ATP and yet not enter the cell cycle [27], although ATP is likely to contribute to the ability of cells to assemble the translation machinery in the Ox phase. What is more important in cell division initiation is nutrient availability and cell size. Budding yeast cells divide once they reach a certain size threshold [28,29], unless the G1/S transition is disabled by certain inhibitors. It may be the case that such inhibitors are active in the Ox phase, and a drop in their activity enables the yeast cells to initiate cell division. It is also possible that a byproduct of the Ox phase promotes the "start" of initiation.

### Msn4

The third most periodic transcription factor that peaks at the end of the R/C phase--just before the Ox phase--is Msn4, which is known to be involved in the diauxic transition and general stress response [30-33]. The fact that Msn4 activity decreases near the time that oxygen levels start to decrease also suggests that Msn4 activity may be changing in response to oxidative stress. Msn4 has also been predicted to show periodic regulatory behavior during the cell cycle, with greatest function during the G1 phase [34]. It is worth noting that Msn4 and Swi6 clustered close together and have extremely similar, but opposite *α*-coefficient profiles. Therefore, Msn4 may have a role in inhibiting the activity of Swi6 and delaying the G1/S transition of the cell cycle under the stress conditions caused by oxidative respiration. The advantage of delaying "start" is that DNA is protected from oxidative damage that would occur if the DNA were replicated during the Ox phase [1,2,35]. However, no previous studies support the connection between Msn4 and Swi6.

#### Possible connection to the diauxic shift

The previously discussed transcription factors are, coincidentally, some of the transcription factors known to be involved in the diauxic shift, the metabolic shift from fermentation to respiration. Hap1, Hap2/3/4/5, Msn2/4 and Yap1 are the transcription factors that mediate the activation of genes encoding antioxidant defenses during this transition [36], and many of these transcription factors were in our list of top transcription factors. There are also other similarities between the diauxic shift and the yeast metabolic cycle. The diauxic shift happens when yeast cells exhaust glucose in the medium and transition into using another carbon source such as ethanol, to produce ATP. In the process, genes encoding mitochondrial biosynthesis and respiratory proteins that had been under glucose repression become derepressed [36]. These are the same events we see happening at the transition of the yeast metabolic cycle from the reductive R/B phase to the oxidative Ox phase. Therefore, it is possible that the long-period yeast metabolic cycle is similar to a cycle of repeated diauxic shifts. During a diauxic shift, the mRNA expression level of more than 1,700 or 27% of all yeast genes changes by more than a factor of two [37]. In addition, 800 or 13% of all yeast genes show periodic expression over the course of the yeast cell cycle [38], adding up to approximately 50% of yeast genes. This number comes close to the 57% of all yeast genes that show periodic expression during the yeast metabolic cycle [2]. On the other hand, one of the main regulators of the diauxic shift is Sip4, which interacts with Snf1, the main regulator of glucose repression [37], and Sip4 is not included in our list of significant transcription factors. Neither is Yap1, another one of the transcription factors involved in oxidative damage during the diauxic shift. These two transcription factors were not significant even when lower thresholds were set during the iterative steps of transcription factor selection. Also, this model does not explain the distinction between genes expressed in the R/B and R/C phases. In summary, although the shift from the respiratory to the oxidative phase of the yeast metabolic cycle show characteristics of the diauxic shift, there are likely other factors at play, as well.

#### Cluster of transcription factors with sharply peaked profiles

Other transcription factors may also be important in regulating the yeast metabolic cycle, based on their periodicity scores, known function, and connectivity in the regulatory network diagram. We will discuss the additional five transcription factors Ace2, Adr1, Bas1, Gcn4 and Spt2 in detail. All five belong to the larger of the two clusters based on cluster analysis and have characteristic spikes in their *α*-coefficient profiles during the Ox phase. Bas1, Gcn4 and Spt2 peak in the Ox phase and show the lowest activity during the R/C phase, while Ace2 and Adr1 have the opposite pattern. All five transcription factors have different functions and contribute in different ways to the patterns of gene expression seen in the yeast metabolic cycle.

### Bas1 and Gcn4

The transcription factors Bas1 and Gcn4 are involved in amino acid and nucleotide metabolism, and they peak in the Ox phase, which is known to be enriched for genes encoding amino acid metabolism, among other functions [2]. Bas1 is involved in histidine, purine and pyrimidine synthesis [39-41] and it is regulated by adenine, a purine derivative which antagonizes its activity [42]. On the other hand, Gcn4 is such is the master regulator of general amino acid control and is involved in regulating 19 of the 20 biosynthesis pathways, and directly or indirectly regulates purine synthesis, organelle biosynthesis, autophagy, glycogen homeostasis, and multiple stress responses [41]. Gcn4 activity is induced by the transcription factor Gcn2, which promotes amino acid storage in yeast vacuoles under glucose starvation [43]. If we assume that glucose starvation conditions are analogous to the respiratory Ox phase of the yeast metabolic cycle, Gcn4p levels would increase during the Ox phase. Then, when metabolism shifts back to fermentation, it would be analogous to returning to the non-starved experiment, where Gcn4p gets degraded, as is the case under normal conditions [44]. Meanwhile, Bas1 activity may follow Gcn4 activity because they are linked by purine. Hypothetically, Gcn4 and Bas1 levels may be tied to the oxidative phase of the yeast metabolic cycle in such a manner.

### Adr1

Glucose repression is regulated in part by Adr1. This transcription factor is required for the expression of the glucose-repressed gene Adh2, peroxisomal protein genes, and genes required for ethanol, glycerol, and fatty acid utilization [45]. This corresponds with the functions enriched in the R/C phase, which include fatty acid oxidation, peroxisome biosynthesis, and glycolysis [2]. There is controversy about what regulates Adr1 biosynthesis and activity. Previous studies have suggested glucose levels, carbon source, or phosphorylation by a functional Snf1 protein kinase [46-48]. We found that Snf1 has low periodicity scores, suggesting that it is not the factor determining Adr1 activity (Rao, data not shown). On the other hand, intracellular glucose levels [11] have a profile very similar to the opposite of the Adr1 *α*-coefficient profile. Snf1 increases the DNA binding activity of Adr1 in the absence of glucose [49], so as glucose levels drop, Adr1 activity would increase even if Snf1 levels remain relatively constant. Thus, genes involved in non-fermentative modes of metabolism would be upregulated at the same time as glucose levels drop. This happens in the R/C phase of the yeast metabolic cycle, as indicated by the peak in Adr1 *α*-coefficients in this phase. It may be the case that increased rates of glycolysis in the R/B phase [2,50] consume intercellular glucose faster than the rate at which glucose enters cells, and in response to a drop in intercellular glucose levels, Adr1 activates alternative metabolic pathways such as fatty acid oxidation to supplement the cell's need for energy.

### Ace2

Ace2, a transcription factor that activates genes in the G1 phase of the cell cycle [51-53], has an *α*-coefficient profile similar to that of Adr1; it peaks in the R/C phase, and has a spike of decreased activity in the Ox phase. This corresponds with the fact that cell division happens during the R/C phase [2], because cells will be in the G1 phase of the cell cycle during the R/B and Ox phases of the yeast metabolic cycle. Ace2 activity is regulated by phosphorylation through a complex signaling network [54-56]. This is associated with Ace2 localizing to the nucleus, where it exhibits its regulatory effect [57]. A transcription factor related to Ace2, Swi5, is regulated in the yeast cell cycle similarly and has a similar expression and *α*-coefficient profile as Ace2 [51,58] (Rao, data not shown). Together, Ace2 and Swi5 regulate the M/G1 transition of the cell cycle [51], and this occurs during the R/C phase of the metabolic cycle, which is where Ace2 peaks.

### Spt2

The transcription factor Spt2 peaks in the Ox phase of the yeast metabolic cycle and is a negative transcriptional regulator associated with transcription elongation, chromatin dynamics, and genome stability [59,60]. Considering that Spt2 peaks during the Ox phase of the yeast metabolic cycle, when DNA is most likely to suffer oxidative damage, it may be that Spt2 plays a role in inhibiting transcription in this phase, although the mechanism of the regulation of Spt2 activity is not clear.

#### Sulfur metabolism and comparison with the short-period metabolic cycle

A previous study on the short-period metabolic cycle [8] suggested that a key regulator of the metabolic cycle is the Cbf1-Met4-Met28-Met31-Met32 complex, which is involved in sulfur assimilation [8,61]. Tu *et al*. [11] observed that several metabolites in the sulfur assimilation pathway exhibit robust oscillations as a function of the long-period yeast metabolic cycle, and strains with a mutation in Cys4, an enzyme in the sulfur pathway, did not undergo metabolic cycles. This suggests that the sulfur metabolic pathway is important in the establishment of the long-period yeast metabolic cycle, as well as the short-period one. However, we did not find any of the transcription involved in the regulation of the sulfur metabolism pathway in the list of top transcription factors. Selecting transcription factors with more lenient thresholds did not result in a list containing any of the Cbf1-Met4-Met28-Met31-Met32 complex, either. Met4 is regulated through general amino acid control by the transcription factor Gcn4 [62]. Since Gcn4 activity is highly periodic over both the long- and short-period metabolic cycles [8] (Table 1), this may explain why the metabolites of the sulfur assimilation pathway oscillate in both types of metabolic cycles. Furthermore, an increase in sulfur metabolism promotes cell division initiation [63], which may explain why cell division is initiated following the Ox phase, which is characterized by an increase in sulfur metabolism [2,11].

#### Accounting for repressor or activator function of transcription factors

The fact that many transcription factors have opposite *α*-coefficient profiles raises the question of whether the profile may be flipped, considering that a transcription factor may be either an activator or a repressor. In some cases, the precise role of a transcription factor is not known. In other cases, the problem may be further complicated by the fact that some transcription factors can act as both a repressor and an activator depending on context [64,65]. Therefore, it may be surprising that the most of the transcription factor *α*-coefficients peak where they are expected to. Hap4, Bas1, Swi6, Msn4 and Gcn4 are known to be transcriptional activators, whereas Spt2 is a transcriptional repressor. In the case of Spt2, the negative role means that an increase in activity results in the repression of genes that it binds to. This must be kept in mind when considering the role of Spt2 or other known repressors in the yeast metabolic cycle, to avoid making an incorrect conclusion.

## Conclusions

Low rates of glucose induce oscillations in yeast metabolism because cells may be maintaining a balance between different pathways of energy production, while most effectively using accumulated resources in each phase. Yeast is unique because it prefers fermentation over respiration, even under aerobic conditions. Under normal aerobic growth conditions and high glucose concentrations, the high rate of fermentation inhibits the synthesis of enzymes involved in respiration; this effect is known as the Crabtree effect [66]. Then, as glucose concentrations decrease, the rate of respiration increases. It is when cells are synchronized by a short period of starvation, as in [2], that we observe oscillations in the form of respiratory bursts. Pathways other than respiration also exhibit oscillations as a function of the yeast metabolic cycle, including cell cycle initiation, fermentative metabolism and other, non-fermentative, modes of reductive metabolism. Studying the regulation of the yeast metabolic cycle requires an understanding of each pathway and their regulation. We chose to highlight only a select group of transcription factors that are involved in regulating the yeast metabolic cycle.

Among the transcription factors regulating the yeast metabolic cycle, Hap1 and Hap4 are directly involved in respiration and are regulated primarily by levels of heme and carbon source, respectively. Therefore, it is likely that these are indeed the main regulators of mitochondrial function in the cycle, as Lelandais *et al*. [10] had proposed. The importance of heme in the yeast metabolic cycle is supported by findings of Tu *et al*. [11]. However, due to the complexity of changes in cellular function during the yeast metabolic cycle, no single transcription factor can be responsible for the observed oscillations, and each transcription factor must be considered in order to form a unified model for the regulation of the yeast metabolic cycle.

In the early Ox phase, we propose that low intracellular glucose concentrations cause cells to progress through the diauxic shift, and oxidative respiration is switched on by the transcription factor Hap1. The oxidative stress induces Msn4, which activates other genes involved in the diauxic shift, as well. Gcn4 activity, which increases during periods of glucose starvation, peaks during the Ox phase, which seems analogous to conditions of glucose starvation regarding cellular function. The peak in Gcn4 indirectly promotes sulfur metabolism by activating the transcription factor Met4 [62]. Energy metabolism is restricted to respiration, and acetyl-CoA and dissolved oxygen levels decrease, while intracellular glucose levels increase.

After oxygen and acetyl-CoA are depleted, respiration ceases, and yeast cells enter the reductive phase of the yeast metabolic cycle. In the R/B phase, Swi6 initiates cell division, possibly due to the burst of sulfur metabolism during the previous phase. In this phase, cell undergo highly glycolytic metabolism to protect DNA from oxidative damage during replication [1,2,35]. Hap4 is responsible for the activation of mitochondrial biogenesis in the R/B phase.

The R/C phase of the yeast metabolic cycle is associated with the transcription factor Adr1. It is involved in glucose repression, and its role is to activate glucose-repressed genes as intracellular glucose levels decrease. This results in the activation of pathways involving fatty acid oxidation, and ethanol and glycerol utilization during the R/C phase.

Further studies should analyze the microarray data set from studies on the short-period oscillations using the methods from this study. Depending on the similarity of the lists of transcription factors regulating the short- and long-period cycles, it would reveal whether the two types of metabolic cycles are fundamentally different or not. It may also be helpful to include other oscillating metabolites, such as glucose, acetyl-CoA, and NADP(H), in the dynamical model.

Wolf *et al*. [67] derived a mathematical model showing that oscillations are induced in the metabolic cycle when the inhibitory effect of H_2_S is added to the glycolysis model. This model supports the importance of the sulfur metabolic pathway for establishing oscillations in the short-period yeast metabolic cycle. A further study could create a mathematical model that includes pathways involving heme synthesis to determine whether the Hap2/3/4/5 complex and regulation of heme biosynthesis are sufficient for inducing oscillations in the long-period yeast metabolic cycle.

Determining how the yeast metabolic cycle is regulated may have implications on other biological cycles and studies on transcription factors, as well. For example, as a function of the mammalian circadian cycle, heme concentrations oscillate [68]. Similarities between the *α*-coefficient profiles of different transcription factors may suggest novel interactions or regulatory mechanisms. For example, Msn4 and Swi6 have very similar, but opposite, *α*-coefficients, suggesting that these may form the connection between oxidative stress and delaying cell cycle initiation during stressful conditions. We speculate that many interesting connections may be discovered by comparing detailed *α*-coefficient profiles from other types of oscillating biological systems, and it would be especially interesting to use the methods of this study to analyze the short-period metabolic cycle.

## Methods

### Estimation of transcription factor *α*-coefficients

The method for calculating transcription factor *α*-coefficients, from [13], uses the assumption that gene expression levels are proportional to the product of the levels of each transcription factor that binds to its promoter. This can be described by the following equation:

(1) $$R_{i}=c\overset{N}{\underset{j=1}{\prod }}b_{ij}^{\alpha_{j}}$$

where *R*_*i *_is the relative expression level of gene *i, b*_*ij *_is the degree to which transcription factor *j *binds to the promoter of gene *i*, and *α*_*j *_is the *α*-coefficient ("activity") of transcription factor *j*. The variable c is a residual constant that the binding factors get scaled by. Taking the logarithm of both sides, Equation 1 becomes:

$$log\left(R_{i}\right)=\overset{N}{\underset{j=1}{\sum }}a_{j}log\left(b_{ij}\right)+c$$

which can be solved using multiple linear regression, as implemented in the MATLAB function robustfit. This function accounts for a constant term in the model by default. For the current problem, the inputs passed to the function are the logarithm of the matrix containing the binding coefficients, and the logarithm of the vector of gene expression data for the time point. The function returns a vector of *α*-coefficients and the regression residual, and these are calculated for each of the 36 time points (Additional File 4). Binding coefficients relating 6229 genes with 203 transcription factors were obtained from Harbison *et al*. [69]. Time series expression data obtained from Tu *et al*. [2] contains gene expression data for 36 time points, starting at 3973 mins after the start of the experiment and ending at 4837 mins. Unlike in the methods of [13], data was not pre-filtered for rows with missing values, because such rows would automatically be filtered out in later steps, i.e. when scoring genes for periodicity.

The MATLAB function robustfit also returns estimates of the standard error for each *α*-coefficient, and these were used to iteratively discard the least significant transcription factors. In each iteration, transcription factors were retained if they had at least nine time points with a p-value below 0.1, and the remaining transcription factors' *α*-coefficients were recalculated. This was repeated until no more transcription factors could be eliminated. Finally, 20 transcription factors remained. Setting different p-value thresholds and number of significant time points such that ~20 transcription factors remain resulted in lists consisting of mostly the same transcription factors.

### Identification of periodic transcription factors

Transcription factors were given a periodicity score based on autocorrelation, which is the cross-correlation of a signal with itself at various time shifts. We first calculated the raw, unscaled cross-correlation sequence of the *α*-coefficients using the MATLAB function xcorr, and found the value at the point where a peak would be expected, if the *α*-coefficient's period were indeed 300 minutes. We normalized this value by dividing it with the cross-correlation at a time shift of zero, in order to obtain the autocorrelation value. The complete MATLAB implementation may be found in Additional File 5. These normalized autocorrelation values were used as periodicity scores, and transcription factors with periodicity scores below a threshold of 0.44 were discarded, such that 13 transcription factors remained. Having this number of transcription factors in the final model maintains a reasonable data-to-parameter ratio [13] and allows our final list to contain several transcription factors of interest, e.g. Hap1 and Hap4, which have been suggested to be regulators of the yeast metabolic cycle by a previous study [10]. By randomly permuting the order of time points for the top 13 transcription factors (*N *= 1000 times for each transcription factor) and recalculating periodicity scores, it was found that they exceeded the threshold of 0.44 in only 0.023% of the random permutations, i.e. the threshold of 0.44 is highly significant (p-value = 2.3 × 10^-4^).

### Computation of amplitudes and phases of *α*-coefficients

Sine waves were fitted to the time-dependent *α*-coefficient curves to estimate the phase and amplitude of each transcription factor, in order to determine the phase of the metabolic cycle they peak in. Indirectly, this would also determine the order of the cellular events taking place in the yeast metabolic cycle. Lelandais *et al*. [10] decomposed profiles into sums of sine waves with different phases. Since these sine waves have the same period, the algorithm reduces to finding the single sinusoidal curve that best fits the data. Therefore, we generated a single sine wave with a period corresponding to one period of the metabolic cycle. The model pattern was shifted in time to find the phase at which the sum of squares of residuals was minimized. After the best phase had been found, the amplitude was varied until the residual sum of squares was again minimized.

The phase of the peaks of *α*-coefficients were also calculated. The data from the 36 time points was averaged to produce 12 time points for each transcription factor, representing the average of *α*-coefficients across the three cycles for which we have data. The maxima of the averaged out *α*-coefficients was found for each transcription factor, and the location along the time axis was converted into radians.

### Further analysis of transcription factor *α*-coefficients

Transcription factors were clustered based on their time-dependent *α*-coefficient profiles using a hierarchical clustering method. Clustering was done using the absolute values of *α*-coefficients, because transcription factors in the same cluster may have opposite *α*-coefficient curves depending on whether they are activators or repressors.

Temporal data for levels of dissolved oxygen in the yeast culture were obtained from [2] and included in the dynamical model. Oxygen levels were normalized and treated as a 14th transcription factor's *α*-coefficients, in order to compare transcription factor *α*-coefficients and oxygen levels side-by-side.

### Determination of time-translation matrices

Computing a transition matrix enables the prediction of transcription factor *α*-coefficients at one time point from the *α*-coefficients at the preceding time point by matrix multiplication:

$$A_{t+1}=TA_{t}.$$

The set of equations can be solved for *T *using:

$$T={\left(A^{T}\cdot A\right)}^{-1}\cdot A^{T}\cdot B$$

where *A *and *B *are the matrix of *α*-coefficients excluding the last and first time points, respectively [70]. The transition matrix was also calculated introducing a constraint to produce only non-negative entries, using the MATLAB function lsqnonneg. A transition matrix with the non-negative constraint may make the resulting model more readily interpretable biologically [13].

The two time-translation matrices were verified for correctness in modeling the dynamical system by multiplying them with the *α*-coefficients of the first time point, and multiplying the resulting vectors with the time-translation matrices again for each successive time point. Model residuals were calculated for each time point by finding the difference between the mean activity of *α*-coefficients calculated using regression and the mean activity of *α*-coefficients calculated using matrix multiplication.

To illustrate the network of transcription factors visually, the transition matrix was converted into a diagram such that the nodes represent transcription factors and edges correspond to the most significant entries in the translation matrix. If connections existed in both directions, only the more significant connection was considered.

### Computational Tools

Algorithms for computing transcription factor *α*-coefficients and their autocorrelation functions, amplitudes and phases, and time-translation matrices were implemented in MATLAB [71]. The network of transcription factors was visualized using the freely available diagram editor yED [72]. *α*-coefficient curves were clustered using TimeClust, a MATLAB-based tool for clustering genes according to their temporal expression profiles [73].

## Authors' contributions

The calculations described in this manuscript were performed by AR. MP provided essential comments and guidance. The manuscript was written by AR and edited by MP. Network layouts, figures and tables were by AR. All authors read and approved the final manuscript.

## Supplementary Material

Additional file 1 **Time-translation matrix with no constraints**. Shaded entries show significant interactions between transcription factors, with a significance threshold of 0.5. Entries shaded darker are positive values, lighter are negative values. Italics indicate that the interaction was not included in the graphical representation of the transition matrix (Figure 7), because an interaction with a greater magnitude exists in the opposite direction. Click here for file

Additional file 2 **Time-translation matrix with constraint to produce non-negative entries**. Shaded entries show significant interactions between transcription factors, with a significance threshold of 0.5. Click here for file

Additional file 3 **Model residuals for two phases of the yeast metabolic cycle**. Residuals were calculated from A) the transition matrix constrained for non-negative entries and B) the non-constrained transition matrix. Click here for file

Additional file 4 **Goodness of fit for multiple linear regression**. Estimates of the square root of residual variance, *σ*, are reported for each time point and were calulated by the MATLAB function robustfit in order to aggregate the residuals into a single measure of predictive power. First, a *σ *estimate (root-mean-square-error) is calculated from ordinary least squares (*σ*_*OLS*_), and a robust estimate of sigma *(*σ_*robust*_*) *is also calculated. The final estimate of *σ *is the larger of σ_*robust *_and a weighted average of *σ*_*OLS *_and *σ*_*robust*_. Note that *σ *is equal to median absolute deviation *(MAD) *of the residuals from their median, scaled to make the estimate unbiased for the normal distribution: *σ *= *MAD*/0.6745. Also shown are the mean of the residuals at each time point. To put residuals on a comparable scale, they are "studentized," that is, they are divided by an estimate of their standard deviation that is independent of their value. Click here for file

Additional file 5 **Autocorrelation function**. MATLAB code for calculating the autocorrelation function of transcription factor α-coefficients. Click here for file
