# Supplementary figures and images for: Regulation of the yeast metabolic cycle by transcription factors with periodic activities
Source: BMC Syst Biol. 2011 Oct 12;5:160. doi: 10.1186/1752-0509-5-160 (PMC3216092; doi:10.1186/1752-0509-5-160)

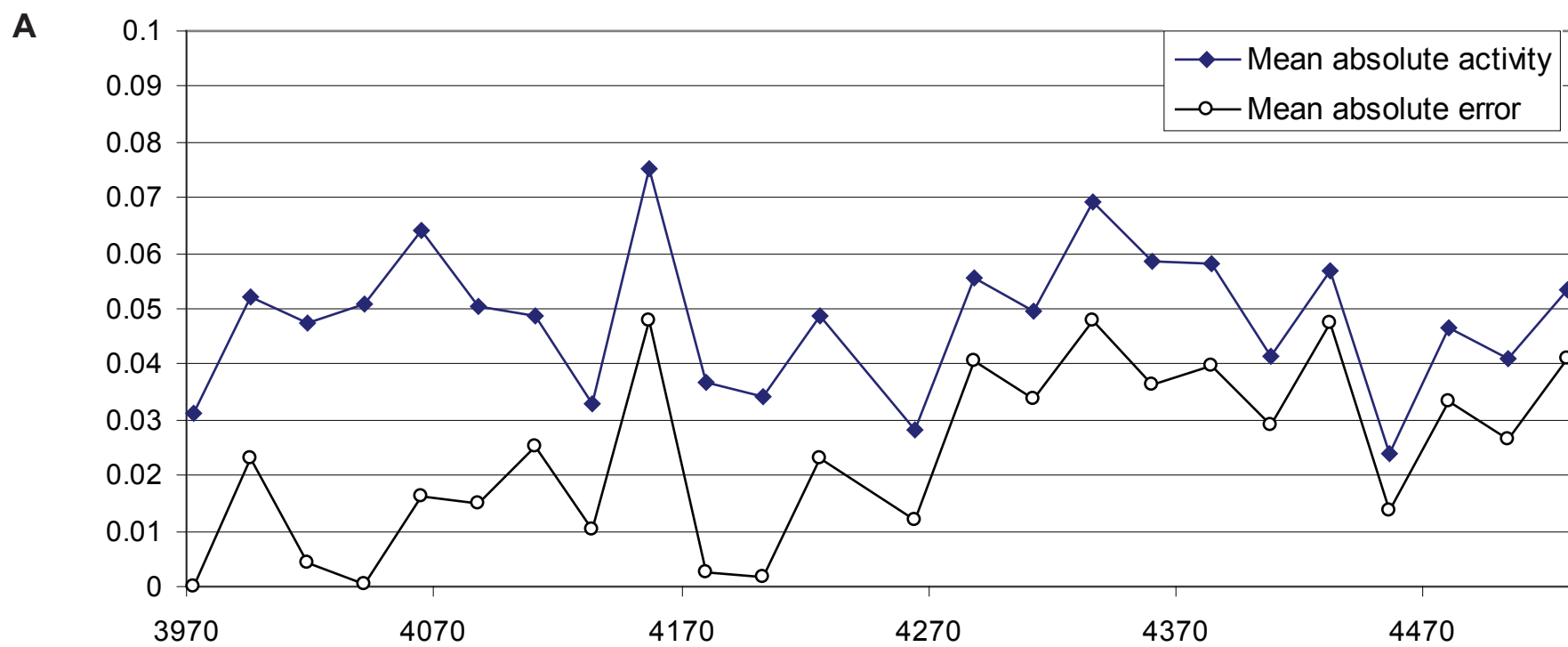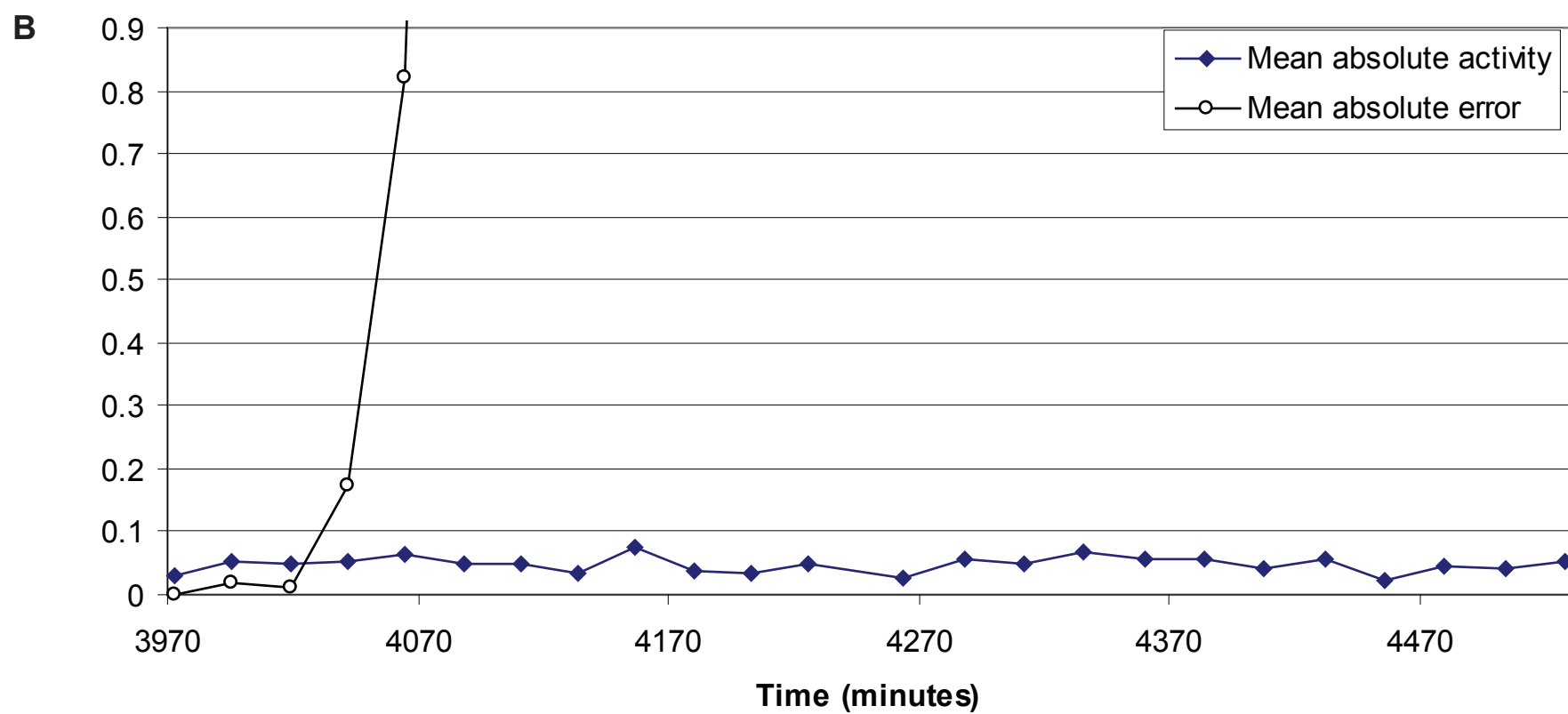

Supplement: Additional file 3 — Model residuals for two phases of the yeast metabolic cycle. Residuals were calculated from A) the transition matrix constrained for non-negative entries and B) the non-constrained transition matrix. [file 1752-0509-5-160-S3.PDF]

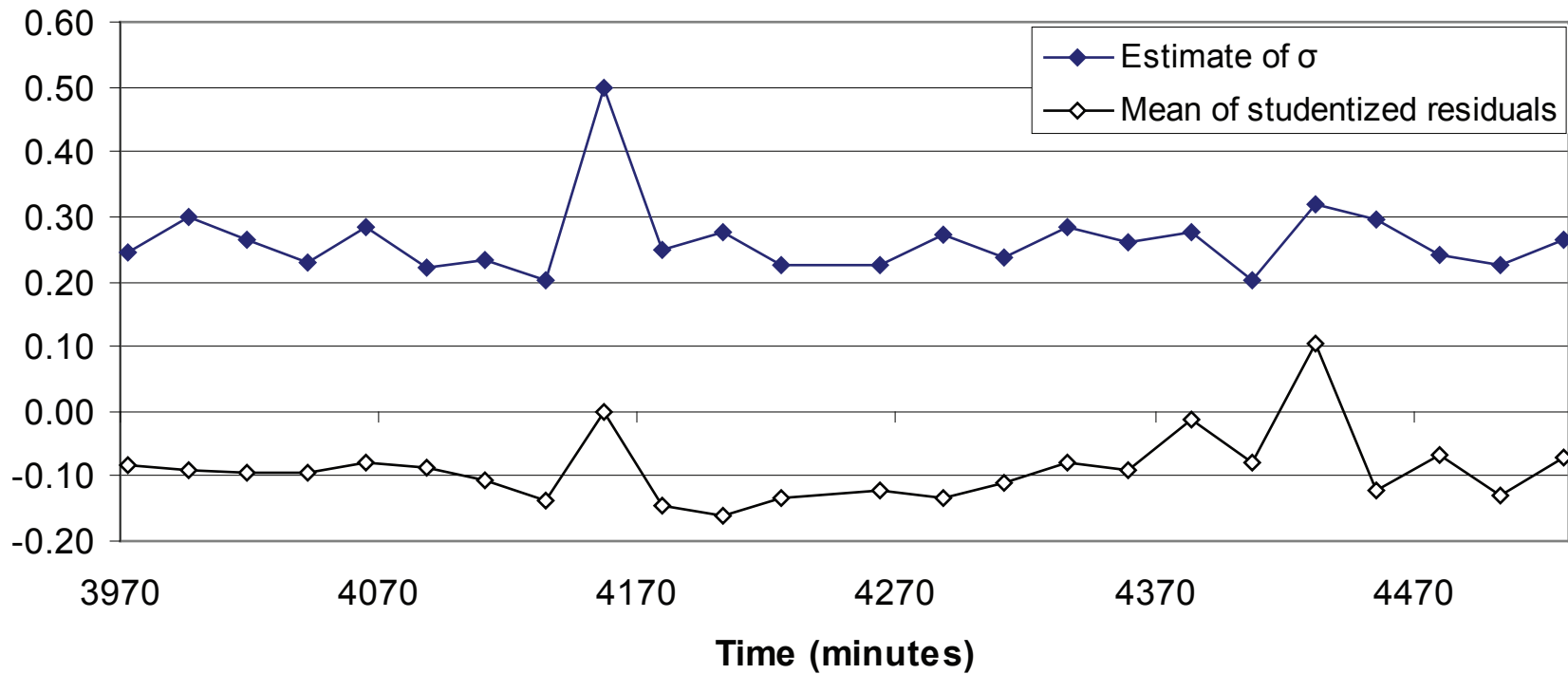

Supplement: Additional file 4 — Goodness of fit for multiple linear regression. Estimates of the square root of residual variance, σ, are reported for each time point and were calulated by the MATLAB function robustfit in order to aggregate the residuals into a single measure of predictive power. First, a σ estimate (root-mean-square-error) is calculated from ordinary least squares (σOLS), and a robust estimate of sigma (σrobust) is also calculated. The final estimate of σ is the larger of σrobust and a weighted average of σOLS and σrobust. Note that σ is equal to median absolute deviation (MAD) of the residuals from their median, scaled to make the estimate unbiased for the normal distribution: σ = MAD/0.6745. Also shown are the mean of the residuals at each time point. To put residuals on a comparable scale, they are "studentized," that is, they are divided by an estimate of their standard deviation that is independent of their value. [file 1752-0509-5-160-S4.PDF]
